# A 3D Print Repository for Plant Phenomics

**DOI:** 10.34133/2020/8640215

**Published:** 2020-11-17

**Authors:** M. Griffiths

**Affiliations:** Noble Research Institute, LLC, 2510 Sam Noble Parkway, Ardmore, OK 73401, USA

## 1. Introduction

In recent years, 3D printing has become a transformative technology for the printing of custom-designed objects outside of traditional manufacturing practices. Printer technology has improved significantly with faster printing speeds, wider choice of print materials, lower machine costs, and free and open-source software. In turn, 3D printing technology is now being adopted across many scientific disciplines for rapid prototyping with emerging utility in plant science. In this article, I present survey results providing an insight into the use of 3D printing in plant science, outline a new online repository for curating plant science-related 3D printed work, and provide a perspective of future adoption for 3D printing in this field.

## 2. Current State of 3D Printing in Plant Science

**Figure 1:**
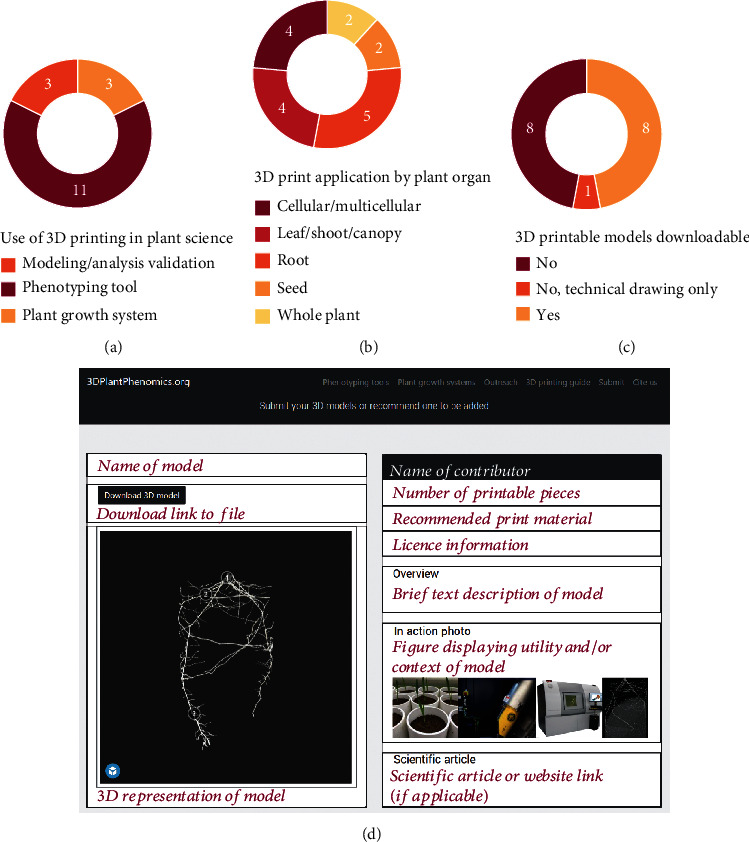
The current state of 3D printing in plant science. (a–c) Peer-reviewed research literature search results of the use of 3D printing in plant science. (d) Webpage layout and example 3D model metadata at 3Dplantphenomics.org, a 3D print repository for plant science.

A literature survey of peer-reviewed scientific research was conducted to determine the status of current 3D printing in plant science. At the time of writing, a total of 17 peer-reviewed papers were found through literature searches online. Three underlying themes for the use of 3D printing in plant science research were apparent: (1) development of novel plant phenotyping tools, (2) development of novel plant growth systems, (3) printing of physical plant models for validation of analysis techniques (Figure 1(a)), and (4) printing plant models as an outreach and teaching aid. The fourth theme is considered a notable use of 3D printing in plant science outside of the peer-reviewed research survey.

The literature survey also provided an insight into the different applications of 3D printing in plant science. The distribution of research was broad from cellular to whole-plant analysis (Figure 1(b)). The majority of 3D prints however were for organ-specific uses with only two studies being for the whole plant which were for plant growth systems [1, 2]. Therefore, 3D printing so far is largely being used to alleviate specific research problems which are likely routine or repetitive tasks and/or benefit from equipment standardization minimizing error. A small majority of 3D print papers focused on plant roots [3–7], which likely reflects the challenge of phenotyping roots and the need for custom solutions.

A surprising statistic from the survey was the number of papers that did not provide downloadable 3D models for readers. Less than half of the surveyed studies included a downloadable model for what was mentioned in the paper (Figure 1(c)). Of those that did, the majority provided the data file in the supplementary data section of the respective journal or on a data repository site. A recent scientific community survey showed that the majority of responders considered the sharing of research data to be a public benefit (66%), and such increases visibility and scientific impact (74%) [8]. However, only 44% of responders believed that open science practices benefit data transparency and reuse. In respect to 3D printing, the act of sharing 3D model files is likely to be of even greater public benefit and scientific impact, as researchers can directly print and utilize the model for their own work. Providing downloadable 3D model designs also allows other researchers to develop upon and improve existing models, adapt models for new plant systems, or create models with new functionality. 3D printing is therefore likely to continue to be a clear example of the benefits of open science with collective improvements in science practice and accelerating research development.

## 3. Online Repository for 3D Printing in Plant Science

As seen in the literature survey results, there is clear early adoption of 3D printing technology in plant science. The results show that the applications of 3D printing in plant research are wide and will likely continue to grow. Despite early utility in plant research, finding such 3D printed work is difficult as no standardization of 3D printing practices or file sharing has yet been established. Many 3D print repositories can be found online for downloading models such as “Thingiverse” and “NIH 3D Print Exchange”, but these websites are general-purpose, have no defined submission standards, and do not provide links to the respective peer-reviewed research. Community curations such as Quantitative-Plant.org have instead proven to be useful for plant image analysis software dissemination and serve as a searchable resource for users [9]. No such repository existed with a focus on 3D printing in plant science. A 3D printing online repository was therefore established at 3Dplantphenomics.org to fulfil this community need.

The aims of this website are to curate a searchable resource of 3D printable models for plant science, to encourage 3D printing dissemination guidelines, and to provide greater exposure to 3D plant science research. 3Dplantphenomics.org will remain up to date and relevant to plant scientists as new peer-reviewed research is published through periodic online literature searches. The website also has an author submission form which will notify the curator with new work to be added. The website currently features four curated lists of 3D models: (1) phenotyping tools, (2) plant growth systems, (3) modeling/analysis, and (4) outreach. These categories will be updated as new functions of 3D printing in plant science arise. Each model list is searchable and can be filtered by typing in a search term such as the model name, author, or plant organ. With selection of a model from the list, an individual model page is presented (Figure 1(d)). This model page provides a 3D WebGL representation of the model, a snapshot description and background of the model, contributing author name and affiliation, model file and file format information, recommendations for print materials, model licence information, and a link to the scientific article and file download where applicable (Figure 1(d)).

A major strength of 3D printing in plant science is that novel methods or analyses are highly reproducible by anyone with a 3D printer or access to a 3D printing online service. For greater scientific transparency and reproducibility, 3Dplantphenomics.org sets recommendations for making 3D model files downloadable if not already available as supplementary data in a research paper or in an online repository. Utilizing a citable data repository such as Zenodo.org or DataDryad.org is advantageous as the model files themselves are provided with a digital online identifier (DOI) link, the authors maintain ownership of their model files, and the files can be updated at a later date with version control if improvements to the model are made.

## 4. Future Perspectives

To attain future global food security, advancements in plant phenomics are urgently needed. 3D printing in plant science is still in its infancy, yet pioneering studies are already showing potential of this technology to advance plant science in multiple ways.

**Figure 2:**
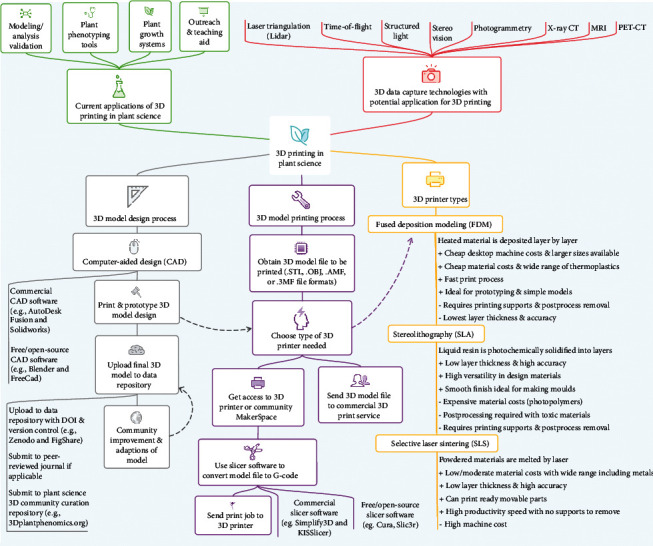
A roadmap of 3D printing in plant science from data collection and design to print.

As 3D printer technology becomes more affordable and accessible worldwide, there is an opportunity to use this to facilitate research coordination and scientific reproducibility. The EcoFAB project has shown great promise at coordinating research across multiple groups with each printing the same 3D printable growth system and following defined protocols with reproducible results [2, 10]. A hurdle for reproducibility of lab protocols is having accessibility to the respective lab equipment. Lab equipment may be too costly to purchase or unavailable at local vendors. With development of more protocols that utilize 3D printed objects, the costs to replicate research will fall and the scientific impact of such work will be greater as a wider audience will benefit from the model file availability. With the ability to conceptualise an idea and then rapidly prototype the model, the freedom of design that a 3D printer provides will speed up innovations in plant science with new phenotyping tools and plant growth systems. Designs that are useful to plant scientists are no longer required to be commercially viable for a vendor to manufacture, as 3D printers can instead print single units out cheaply and quickly. As 3D printing technology in plant science matures, an open-source approach will be important for collective improvement of models and for adapting useful models for different plant systems (Figure 2).

Nondestructive digitization of plants is an expanding research area with 3D image capture of roots, shoots, and canopies being used to visualise and understand plant developmental processes and how plants interface with the environment (Figure 2). Volumetric scans from technologies such as X-ray microcomputed tomography (*μ*CT) and magnetic resonance imaging (MRI) are helping to conceptualise complex plant anatomic structures that are otherwise hidden [11, 12]. Lidar and photogrammetric setups for proximal sensing are likely to grow rapidly over the next few years as the image analysis techniques improve and as camera prices fall [13, 14]. 3D printing of plants is an important means of providing ground truth validation to analysis models [7] and is also proving to be a great STEM (science, technology, engineering, and mathematics) teaching and outreach aid [15].

To facilitate the adoption and use of 3D printing technology in plant science, an online repository has been set up at 3Dplantphenomics.org. Curation of 3D printed models on this site will hopefully be a useful resource to plant scientists, provide greater exposure to 3D plant science research, and encourage dissemination guidelines.
